# Role of NO and S-nitrosylation in the Expression of Endothelial Adhesion Proteins That Regulate Leukocyte and Tumor Cell Adhesion

**DOI:** 10.3389/fphys.2020.595526

**Published:** 2020-11-13

**Authors:** Gaynor Aguilar, Tania Koning, Pamela Ehrenfeld, Fabiola A. Sánchez

**Affiliations:** ^1^Instituto de Inmunología, Facultad de Medicina, Universidad Austral de Chile, Valdivia, Chile; ^2^Instituto de Anatomía, Histología y Patología, Facultad de Medicina, Universidad Austral de Chile, Valdivia, Chile; ^3^Centro Interdisciplinario de Estudios del Sistema Nervioso, Universidad Austral de Chile, Valdivia, Chile

**Keywords:** nitric oxide, S-nitrosylation, leukocyte adhesion, tumor cell adhesion, inflammation

## Abstract

Leukocyte recruitment is one of the most important cellular responses to tissue damage. Leukocyte extravasation is exquisitely regulated by mechanisms of selective leukocyte-endothelium recognition through adhesion proteins in the endothelial cell surface that recognize specific integrins in the activated leukocytes. A similar mechanism is used by tumor cells during metastasis to extravasate and form a secondary tumor. Nitric oxide (NO) has been classically described as an anti-inflammatory molecule that inhibits leukocyte adhesion. However, the evidence available shows also a positive role of NO in leukocyte adhesion. These apparent discrepancies might be explained by the different NO concentrations reached during the inflammatory response, which are highly modulated by the expression of different nitric oxide synthases, along the inflammatory response and by changes in their subcellular locations.

## Leukocyte Adhesion and Nitric Oxide (NO)

Inflammation involves the interplay of multiple biologic components, among which endothelial cells are key players. Endothelial cells orchestrate leukocyte transmigration to injured tissues by up-regulating adhesion proteins on their surface to bind integrins in leukocytes (Ley et al., 2007; Fan and Ley, 2015; Kreuger and Phillipson, 2016). Initial contact and rolling steps are initiated by endothelial cell leukocyte adhesion molecule-1 (ELAM-1, E-selectin) and P-selectin, which are expressed in the endothelium and bind to L-selectin, PSGL-1, CD44, CD43, and ESL-1 in the leukocyte. During the rolling phase, the interactions between leukocytes and endothelial selectins reduce leukocyte velocity and facilitate their adhesion to endothelium. Selectin proteins have a high degree of association/dissociation with their leukocyte ligands, which allows contact between the endothelial cell and leukocytes and provides enough time and proximity for other adhesion molecules to establish strong bonds between both cells (Sperandio, 2006; Garrido-Urbani et al., 2008; Tvaroška et al., 2020). Firm leukocyte adhesion to endothelial cells is mediated by vascular cell adhesion molecule 1 (VCAM-1) and intercellular adhesion molecule 1 (ICAM-1) that bind to leukocytes integrin’s VLA-4, LFA-1, and Mac-1 (Greenwood et al., 2003). Once the leukocytes are attached to the endothelium, they flatten by contacting the endothelium at varying distances, probably to reduce their exposure to blood flow forces and collisions with circulating blood cells, and subsequently they initiate their trans-endothelial migration (Ley et al., 2007; Leick et al., 2014).

Nitric oxide (NO) is a physiological messenger that regulates many cellular functions, such as vasodilation, angiogenesis, vascular permeability, neurotransmission, cell migration, immune response, cell proliferation and apoptosis (Tuteja et al., 2004; Nagy et al., 2007; Koriyama and Furukawa, 2018; Ehrenfeld et al., 2019; López-Sánchez et al., 2019). NO is produced in the organism by three different nitric oxide synthases: endothelial (eNOS, mainly expressed in endothelium), inducible (iNOS, expressed primarily on the immune system and endothelial cells) and neuronal (nNOS, expressed in the nervous system) (Förstermann and Sessa, 2012). NO produced by these isoforms activates two main signaling pathways: (1) soluble guanylate cyclase – protein kinase G (GC1-PKG) and (2) S-nitrosylation, which is the modification induced by NO in free-thiol cysteines in proteins to form S-nitrosothiols. S-nitrosylation regulates interactions between proteins, phosphorylation and intracellular trafficking (Stamler et al., 1992; Huang et al., 2005; Marín et al., 2012; Guequén et al., 2016).

The first studies addressing the role of NO in leukocyte adhesion used different inhibitors of NO production like L-NG-monomethyl arginine (L-NMMA) or N omega-Nitro-L-arginine methyl ester (L-NAME) to observe the effect on the basal leukocyte adhesion (in the absence of inflammatory stimulation). These experiments showed an increased basal leukocyte adhesion *in vivo* in different animal preparations and endothelial cell cultures *in vitro* (Kubes et al., 1991; Arndt et al., 1993; Ma et al., 1993; Tsao et al., 1994). The opposite approach, to elevate the NO concentration by the use of NO donors, prevented leukocyte adhesion and infiltration depending on NO level (Johnson et al., 1990; Kubes et al., 1991; Kubes and Granger, 1992). Studies using intravital microscopy in knockout (KO) animals for eNOS and nNOS corroborated these results showing increased leukocyte adhesion relative to control animals in the mesentery (Lefer et al., 1999). These observations and those from other laboratories led to the well-established concept that, in healthy endothelium, there is a physiological constitutive level of NO produced by eNOS that confers anti-adhesive and anti-inflammatory properties to the endothelial cell membrane and plays a critical role in preventing leukocyte adhesion (Kubes et al., 1991; Tsao et al., 1994). On the other hand, when the endothelium is stimulated with pro-inflammatory agonist, the effects of NO have not been completely consistent. There is a vast body of evidence showing an inhibitory effect of NO on stimulated leukocyte adhesion (De Caterina et al., 1995; Liu et al., 1998; Baatz and Pleyer, 2001; Lelamali et al., 2001; Lo et al., 2001; Jiang et al., 2005; Shelton et al., 2008); while other reports demonstrate either no effect of inhibition of NO on leukocyte adhesion (Hickey et al., 2001; Shelton et al., 2008) or promotion – mediated by NO – of leukocyte adhesion in response to challenge with cytokines (Bessa et al., 2002; Joussen et al., 2002). These discrepancies may be due to several differences in experimental approaches including timing and duration of the application of agonist/antagonist. We review here how NO regulates leukocyte adhesion by acting on different mechanisms that regulate surface expression of adhesion proteins on the endothelium such as: (a) through transcriptional regulation; (b) through non-transcriptional regulation, including traffic of vesicles to plasma membrane and clustering of adhesion proteins normally expressed in the endothelium. NO can also regulates integrin and protein expression and/or affinity on leukocytes (Kubes et al., 1991; Banick et al., 1997; Mitchell et al., 1998; Thom et al., 2013; Bhopale et al., 2015); however, given the enormous big of data on the topic, this review will focus only in the NO effects on the adhesion protein expression on the endothelium.

### Transcriptional Regulation of Adhesion Proteins and NO

**FIGURE 1:**
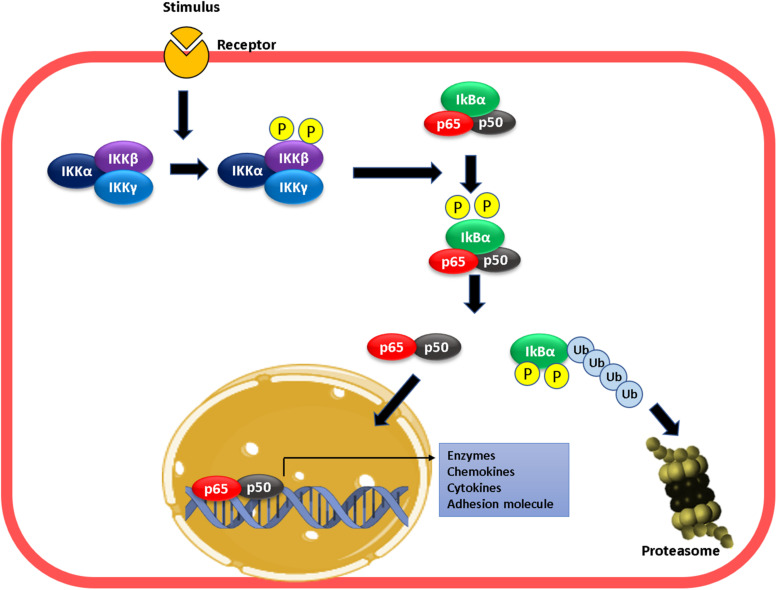
Mechanism of NF-kB activation. Proinflammatory stimulation leads to the phosphorylation of IKKβ leading to phosphorylation of IKBα in NF-κB. This phosphorylation result in the ubiquitination and degradation of IκB-α in the proteasome. At this moment, the dimer p50–p65 enters into the nucleus, binds to the targets gene promoter (κB sites), and activates transcription.

The effects of NO on leukocyte adhesion are related to transcriptional regulation of adhesion proteins expression on the endothelium (De Caterina et al., 1995; Khan et al., 1996; Liu et al., 1998; Lefer et al., 1999; Waldow et al., 2006; Buckanovich et al., 2008; Carreau et al., 2011; Stojak et al., 2018). Transcriptional regulation adhesion proteins, as well as numerous proinflammatory genes, is under the control of NF-κB (Springer, 1990; Collins et al., 1995; Ledebur and Parks, 1995; Pierce et al., 1997; Qian and Fulton, 2012), a dimeric protein formed by any of the members of the Rel family (p50, p65 or Rel-A, p52, c-Rel, and RelB) (Thanos and Maniatis, 1995; Marshall and Stamler, 2001). The classic form of NF-κB is the p50–p65 heterodimer that is kept inactive in the cytosol via interaction with the inhibitory protein IKBα (Thanos and Maniatis, 1995; Marshall and Stamler, 2001; Hayden and Ghosh, 2008; Sha and Marshall, 2012). Activation of cytokine receptors promotes the phosphorylation, ubiquitination, and proteasomal degradation of IKBα mediated by the IKKβ subunit of the IKK complex, formed by IKKα, β, and γ, which results in translocation of the p50–p65 heterodimer to the nucleus to bind target DNA sites, and activate gene transcription (Marshall and Stamler, 2001; Hayden and Ghosh, 2008; Sha and Marshall, 2012; Figure 1). Activation of the NF-κB pathway leads to *de novo* synthesis of high levels of messenger RNA for E-selectin, P-selectin, ICAM-1, and VCAM-1, which induces an increase in protein expression of these proteins in activated endothelial cells, and enhances the adhesion of leukocytes on the cell surface (Whelan et al., 1991; Iademarco et al., 1992; Van De Stolpe et al., 1994; Pan and McEver, 1995; Xia et al., 2001; Mussbacher et al., 2019).

**FIGURE 2:**
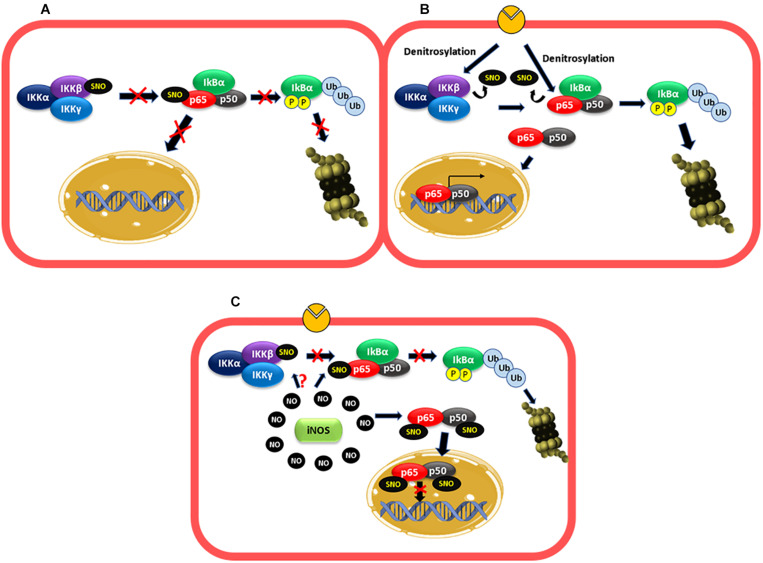
NF-κB is regulated by S-nitrosylation. **(A)** Basal conditions: basal S-nitrosylation of IKKβ and p65 blocks IKBα phosphorylation and degradation proteasomal and the entry of the dimer p50–p65 to the nucleus, keeping NF-κB inactivated. **(B)** Short inflammatory stimulation: under stimulation, there is a decrease in the S-nitrosylation levels of IKKβ and p65 favoring IKBα phosphorylation and proteasomal degradation and the consequent entry of the dimer p50–p65 to the nucleus to increase adhesion protein transcription among others. **(C)** Long stimulation: at long periods of exposure to proinflammatory agents, high levels of NO-produced by iNOS induces S-nitrosylation of p65 and p50, which inhibit the binding of the dimer to the DNA. IKKβ might also be S-nitrosylated contributing to inactivate NF-κB.

Early investigations demonstrated that inhibition of NO production activated NF-κB and protein adhesion expression, whereas NO donors had the opposite effect (De Caterina et al., 1995; Khan et al., 1995). These effects were not mediated by the GC1-PKG pathway and appeared to depend strictly on NO concentration, suggesting that S-nitrosylation might be the operating mechanism (De Caterina et al., 1995; Lee et al., 2002; Waldow et al., 2006). Later, it was demonstrated that there is a basal S-nitrosylation of IKKβ that prevents IKBα phosphorylation and degradation, keeping NF-κB inactive (Figure 2A; Reynaert et al., 2004). Basal S-nitrosylation of p65 has also been reported in respiratory epithelium (A549 cells) and lung tissue (Kelleher et al., 2007, 2011). Inhibition of NOS activity reduced nitrosylation of IKKβ, leading to NF-κB activation in Jurkat T cells (Reynaert et al., 2004). Even though the role of basal S-nitrosylation has not been investigated, studies using cytokine-stimulated cells indicate that S-nitrosylation of p65 blocks the binding of NF-κB to DNA (Kelleher et al., 2007, 2011). Therefore, it is possible that basal S-nitrosylation of p65 contributes to keeping NF-κB inactivated. We postulate that the dynamics of S-nitrosylation cellular levels may be key to activation/inactivation of NF-κB.

After short inflammatory stimulation (10 min–2 h) with TNF-α or LPS, the S-nitrosylation levels of IKKβ and p65 decrease and NF-κB is activated (Reynaert et al., 2004; Kelleher et al., 2011, 2014; Figure 2B). At long times of exposure (6h, LPS), p65 is S-nitrosylated again with the consequent NF-κB inactivation (Kelleher et al., 2007, 2011). Studies using iNOS KO mice revealed that the high levels of NO-induced by this enzyme mediated this effect since genetic deletion of iNOS in mice blocks the recovery of S-nitrosylation of p65, and NF-κB activation is maintained along with prolonged inflammation in a model of LPS-induced lung inflammation (Kelleher et al., 2011). In addition, in a colitis model in iNOS KO mice, inflammation and elevated MPO activities persisted at 2 weeks compared to control mice, which improved colitis and decreased MPO activity (Vallance et al., 2004). iNOS also mediates S-nitrosylation of p50, which contributes to inhibiting NF-κB DNA binding (Matthews et al., 1996; DelaTorre et al., 1998; Marshall and Stamler, 2001). Even though it has not been reported, the high levels of NO-induced by iNOS might also re-nitrosylate IKKβ contributing to NF-κB inactivation (Figure 2C).

Although the evidence described above strongly points to a physiological role for iNOS in the inactivation of NF-κB, other report demonstrated that higher concentrations of NO – beyond those reached by iNOS expression – are required for NF-κB inactivation by S-nitrosylation of IKKβ and p65 (Qian and Fulton, 2012). It is important, however, to note that the report by Qian and Fulton used endothelial cells transfected with iNOS and not physiological iNOS induction by stimulation with pro-inflammatory cytokines.

**FIGURE 3:**
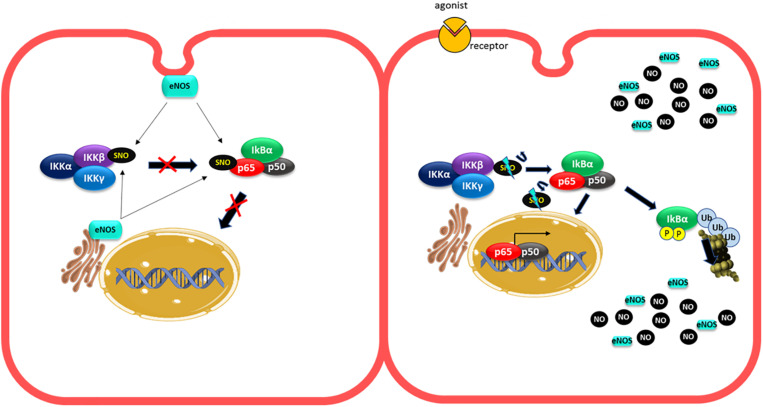
Changes in eNOS localization under short inflammatory stimulation can activate NF-κB. In unstimulated cells, the basal production of NO generated by eNOS (located at the Golgi and caveolae) provides basal S nitrosylation of IKKβ and p65 to maintain an anti-adhesive phenotype **(left pannel)**. Under short-term proinflammatory conditions, eNOS is activated and changes in its location might decrease NO levels around IKKβ and p65, decreasing their S-nitrosylation levels leading to NF-κB activation **(right pannel)**.

In strong contrast to the results described above, other investigations demonstrated that inhibition of NO production did not increase adhesion protein expression induced by cytokines or leukocyte adhesion to endothelial monolayers (De Caterina et al., 1995). Furthermore, some reports have demonstrated that NO promotes protein adhesion expression. In diabetic rats, a high retinal leukocyte adhesion was observed, which correlated with a rise in NO and ICAM-1 levels. Pharmacological inhibition of NOS with L-NAME also reduces leukocyte adhesion (Joussen et al., 2002). In human vascular aortic smooth muscle cells (HASMCs), LPS treatment by 24 h increased ICAM-1 expression. Inhibition of NOS with L-NAME, prior to LPS administration, inhibited this effect, demonstrating that NO is required for enhancing ICAM-1 expression under this experimental conditions (Heo et al., 2008). In addition, the stimulation of rat microvascular endothelial cells with VEGF, that activates eNOS, increased ICAM-1 expression in 30 min, suggesting that eNOS regulates ICAM-1 transcription (Radisavljevic et al., 2000). The fact that eNOS is involved suggests that low NO concentrations but higher than basal might stimulate adhesion protein transcription. In fact, in HUVECs cells, NO donors increased ICAM-1, VCAM-1, and ELAM-1 expression. This effect was dependent on the NO donor concentrations used, with low concentrations (10 and 50 μM) inducing adhesion protein expression, whereas higher levels (250 and 500 μM) did not cause protein adhesion expression (Sektioglu et al., 2016). Microarray analysis of HUVEC treated with NO donors at low concentration or TNF-α demonstrated that 473 genes were upregulated, including VCAM-1 and E-selectin. The effect of NO donor on protein adhesion expression was dependent on NF-κB activation, because the effect was blocked by the NF-κB inhibitor BAY11-7082. These results coincide with earlier studies demonstrating that low NO concentration improved NF-κB activity induced by TNF-α, whereas higher concentrations inhibited NF-κB activity (Umansky et al., 1998). These low NO doses (but still higher than basal), might be achieved by eNOS activation, as many pro-inflammatory agents induce rapid eNOS activation (Marín et al., 2012; Guequén et al., 2016). The precise mechanism by which exogenous administration of low concentrations of NO activate NF-κB dependent protein adhesion expression in endothelial cells remains to be explored. A possible explanation could be given by the changes in eNOS localization under inflammatory stimulation (Figure 3). In non-stimulated cells, basal NO production generated by eNOS (located in the Golgi and caveolae) (Feron and Balligand, 2006; Zhang et al., 2006) maintains an anti-adhesive phenotype through basal S-nitrosylation of IKKβ and p65 (Matsushita et al., 2003; Feron and Balligand, 2006; Zhang et al., 2006). Under short-time stimulated conditions, eNOS is activated and change its localization (Sánchez et al., 2006, 2011; Marín et al., 2012), which might reduce S-nitrosylation of IKKβ and p65 activating NF-κB. Additionally, eNOS-induced NO might activate protein adhesion expression by mechanisms still unknown. At extended times of inflammatory stimulation, the high levels of NO induced by iNOS activation will inhibit NF-κB by enhancing S-nitrosylation of IKKβ, p65 and additional S-nitrosylation of p50. Thus, low concentrations of NO achieved by eNOS activation and short exposure times to agonists increase adhesion protein expression, whereas higher NO concentrations achieved by iNOS stimulation and longer times of stimulation will inhibit protein adhesion expression (Umansky et al., 1998; Sektioglu et al., 2016).

### Non-transcriptional Regulation of Adhesion Proteins and NO

Many pro-inflammatory agents induce a fast leukocyte adhesion observed within minutes of exposure to some pro-inflammatory agents (Dillon et al., 1988; Sugama et al., 1992; Javaid et al., 2003) that cannot be explained by transcriptional regulation. This fast adhesion may be explained by mechanisms such as vesicle traffic and clustering (Javaid et al., 2003; Ley et al., 2007; Lowenstein, 2007; Barreiro et al., 2008; Setiadi and McEver, 2008; Liu et al., 2011).

#### Vesicle Traffic

Adhesion proteins are stored in vesicles in endothelial cells that are transported to the plasma membrane after inflammatory stimulation. ELAM-1, for instance, is stored in intracellular secretory granules of endothelial cells and is rapidly expressed on the cell surface following degranulation, within minutes of exposure to activating agents such as thrombin, histamine, or phorbol esters (Geng et al., 1990; Larsen et al., 1992). The same is true for P-selectin, which is usually stored in Weibel-Palade bodies (WPB) (Weibel and Palade, 1964; Metcalf et al., 2008). In the case of VCAM-1, evidence indicates that after TNF-α treatment of endothelial cells, vesicular transport from an intracellular pool to the cell surface is increased (MacKesy and Goalstone, 2011).

#### Clustering

In basal conditions without inflammatory stimulation, low levels of adhesion proteins are present in an inactive state in the endothelial plasma membrane (Barreiro et al., 2002, 2008; Javaid et al., 2003). The lateral association of adhesion proteins, a process known as clustering, also regulates their adhesive capacity (Javaid et al., 2003; Barreiro et al., 2008; Setiadi and McEver, 2008; Liu et al., 2011). Through clustering, cell surface expression of adhesion proteins does not change; instead, they are regrouped and interact with cytoskeletal proteins to increase their avidity for leukocyte integrins (Javaid et al., 2003; Liu et al., 2011). This affinity change process occurs at short times of stimulation (minutes) and allows a fast response under inflammatory stimulation. In the case of ELAM-1, clustering is promoted by association to lipid rafts and caveolae at the plasma membrane (Tilghman and Hoover, 2002a,b; Setiadi and McEver, 2008). P-selectin clustering is mediated by interactions of the cytoplasmic domain of P-selectin with clathrin-coated pits (Setiadi et al., 1998). Clustering of ICAM-1 and VCAM-1 is promoted by association with tetraspanins, which are small transmembrane proteins able to associate laterally with different proteins at the plasma membrane (Barreiro et al., 2008). ICAM-1 clustering is also regulated by protein kinase C zeta (PKCz), that under stimulation with TNF-α, is activated and translocated to the plasma membrane to phosphorylate ICAM-1 either directly or through Src activation (Javaid et al., 2003; Liu et al., 2011).

#### NO Role in Vesicle Transport and Clustering

**FIGURE 4:**
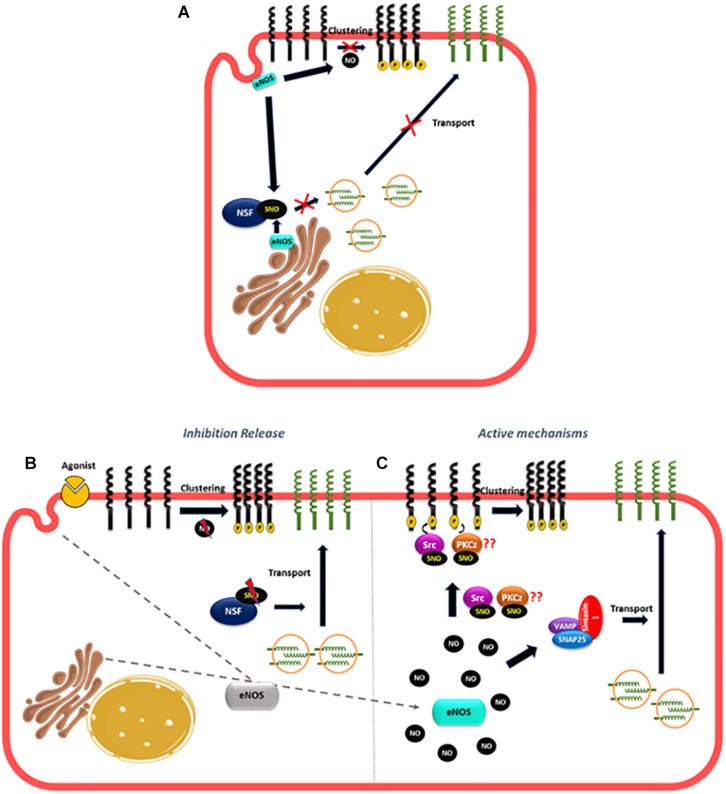
Proposed mechanism of NO role in the fast leukocyte adhesion. **(A)** In unstimulated cells, the basal NO production generated by eNOS located to the Golgi and caveolae maintain an anti-adhesive state in the endothelium by basal NSF-S-nitrosylation that inhibits the translocation of vesicles containing adhesion molecules to the plasma membrane and by blocking the clustering of adhesion molecules like ICAM-1. **(B)** After short inflammatory stimulation, eNOS redistribution, might put NO away from NSF, diminishing NSF-S-nitrosylation to stimulate the transport of vesicles to the plasma membrane. At the same time, the change in eNOS localization might also lower NO levels close to ICAM-1 to promote ICAM-1 clustering. **(C)** At the same time, the movement of eNOS to different subcellular locations will increase NO levels at those locations, which may active the SNAREs complexes (VAMP, SNAP25, and syntaxin) stimulating traffic of vesicles; NO can also induce S-nitrosylation of Src and PKCz to promote ICAM-1 phosphorylation and clustering.

Many reports have demonstrated a NO role in anterograde and retrograde transport (Iwakiri et al., 2006; Lowenstein, 2007; Lee et al., 2009; Marín et al., 2012). N-ethylmaleimide sensible factor (NSF) is an ATPase that modulates vesicular transport (Söllner et al., 1993; Morgan and Burgoyne, 2004) and studies *in vitro* and *in vivo* have shown that there is a basal NSF-S-nitrosylation mediated by eNOS that inhibits the translocation of WPB containing P-selectin to the plasma membrane contributing to the anti-adhesive properties of basal NO production in the endothelium (Matsushita et al., 2003; Lowenstein, 2007). Basal eNOS activity also contributes to the anti-adhesive properties of the endothelium by inhibiting ICAM-1 clustering as it has been demonstrated that eNOS inhibition led to ICAM-1 clustering mediated by Src phosphorylation in endothelial cells *in vitro* and *in vivo* (Xu et al., 2013; Gao et al., 2018). These observations can account for the fast leukocyte adhesion observed when basal NO production is inhibited in the absence of inflammatory stimulation (Kubes et al., 1991; Ma et al., 1993; Gao et al., 2018) but do not explain the fast leukocyte adhesion observed under inflammatory stimulation (Dillon et al., 1988; Sugama et al., 1992; Javaid et al., 2003). Changes in eNOS localization might again explain this effect. In non-stimulated cells, basal NO production generated by eNOS located to the Golgi and caveolae (Feron and Balligand, 2006; Zhang et al., 2006) will maintain an anti-adhesive phenotype in part by basal S-nitrosylation of NSF and also by inhibiting ICAM-1 clustering (Feron and Balligand, 2006; Zhang et al., 2006; Lowenstein, 2007; Mukhopadhyay et al., 2008; Gao et al., 2018; Figure 4A). In fact, only wild type eNOS (located at Golgi and caveolae) induce NSF-S-nitrosylation and inhibit vesicular transport but not eNOS located at the nucleus (Iwakiri et al., 2006). Furthermore the treatment of endothelial cells with monocrotaline pyrrole (MCPT), a drug that blocks the association of NSF with eNOS at Golgi and prevents eNOS localization in caveolae inhibits NSF-S-nitrosylation (Mukhopadhyay et al., 2008; Lee et al., 2009). After short inflammatory stimulation, eNOS redistribution from caveolae and Golgi to cytosol (Sánchez et al., 2006, 2011; Marín et al., 2012), might reduce S-nitrosylation of NSF, to stimulate the release of WPB bodies (Figure 4B). At the same time, the change in eNOS localization might also diminish NO levels close to ICAM-1 in the plasma membrane to stimulate ICAM-1 clustering. On the other hand, the movement of eNOS to different subcellular locations will increase NO levels at those locations, which may actively stimulate traffic of vesicles, stimulating for instance the formation of complexes that promote transport to the cell surface such as VAMP/SNAP-25/syntaxin-1a and/or VAMP-3/syntaxin-2 that have been demonstrated to be stimulated by NO and promote synaptic vesicle exocytosis and release of secretory granules in neurons and platelets, respectively (Meffert et al., 1996; Randriamboavonjy et al., 2004; Figure 4C). Interestingly, NSF-S-nitrosylation has also been reported to stimulate protein transport to the cell surface of AMPA receptors and VSVG protein in neurons and epithelial cells, respectively (Huang et al., 2005; Iwakiri et al., 2006) unlike its inhibitory role in endothelial cells. Considering that NSF has 8 cysteines that can be S-nitrosylated (Matsushita et al., 2003), it is possible that different degrees of S-nitrosylation may also play a role in transport to the plasma membrane. Basal S-nitrosylation of NSF will inhibit transport, whereas decrease or elevation in S-nitrosylation might stimulate traffic. Besides traffic, an elevation in NO, might also stimulate ICAM-1 clustering since Src that promote ICAM-1 phosphorylation is activated by S-nitrosylation in another cellular context (Rahman et al., 2010). PKCζ, that also participate in ICAM-1 clustering (Javaid et al., 2003; Matsushita et al., 2003; Liu et al., 2011) might be also activated by NO and S-nitrosylation since N-ethylmaleimide that blocks the thiol groups decreases the activity of PKCζ (Kikkawa et al., 1987) and a PKCζ synthetic peptide containing the activation site is S-nitrosylated *in vitro* (Balendran et al., 2000; Wang et al., 2008).

Thus, the combined effects of releasing the inhibition induced by basal NO on vesicular transport and clustering, plus the active stimulation by NO of pathways that increase vesicular transport and clustering, will induce leukocyte adhesion at short times of simulation.

## No and Tumor Cell Adhesion

There is considerable evidence demonstrating a dual role of NO and S-nitrosylation in cancer risk and metastasis (Burke et al., 2017; Rizza and Filomeni, 2018; Ehrenfeld et al., 2019; Hays and Bonavida, 2019; Holotiuk et al., 2019; Somasundaram et al., 2019). The available evidence demonstrates a positive correlation between NO biosynthesis, tumor development and degree of malignancy in a variety of cancers (breast, pancreatic, liver, cervical, ovary, melanoma, nasopharyngeal, stomach, colon, lung, oral, esophagus, glioma, and prostate cancer) (Monteiro et al., 2015; Vanini et al., 2015; Burke et al., 2017; Thomas and Wink, 2017; Somasundaram et al., 2019). On the other hand, in animal studies, inhibition or genetic deletion of eNOS or iNOS can inhibit, have no effect, or even increase primary tumor growth and metastasis depending on the type of cancer (Wang et al., 2001, 2003; Gratton et al., 2003; Heinecke et al., 2014; Granados-Principal et al., 2015; McCrudden et al., 2017; Kij et al., 2018; Romagny et al., 2018; Flaherty et al., 2019). These discrepancies probably reflect the fact that NO effects strongly depend on its concentration, duration of exposure, location and activity of NOS isoforms, tumor type, its microenvironment and sensitivity to NO (Ridnour et al., 2006; López-Sánchez et al., 2019; Somasundaram et al., 2019). In the tumoral microenvironment, tumor cells express iNOS, eNOS, and nNOS, depending on tumor type and stage, endothelial cells express eNOS and iNOS whereas tumor-associated stromal fibroblasts and immune cells express iNOS (Fukumura et al., 2006; Monteiro et al., 2015; López-Sánchez et al., 2019). Therefore, the results can be conflicting depending on the experimental set-up and the cell type being investigated.

Despite remarkable developments in cancer therapeutics, metastasis is still closely associated with high mortality rates in cancer patients. Metastasis occurs when tumor cells separate from the primary tumor, enter the bloodstream (or lymph) and travel to remote organs to form a secondary tumor. One of the final events in metastasis is the extravasation of cancer cells across the endothelial barrier (Talmadge, 2010; Sökeland and Schumacher, 2019). Cytokines and other factors produced by the primary tumor, circulating tumor cells and cells in the metastatic microenvironment promote binding between tumor cells and endothelial cells (Wolf et al., 2012; Reymond et al., 2013; Strilic and Offermanns, 2017). Initial insights into tumor cell extravasation were derived from studies of leukocyte extravasation, resulting in the widely accepted concept that cancer cell and leukocyte extravasation – although different – share many similarities (Strell and Entschladen, 2008; McDowell and Quail, 2019; Sökeland and Schumacher, 2019). Increased cell surface expression of adhesion proteins ELAM-1, ICAM-1, VCAM-1, and P-selectin in the endothelium mediate direct adhesion to CD44, mucin-1 and CD24 in breast tumor cells (Aigner et al., 1998; Rahn et al., 2005; Geng et al., 2012; Shirure et al., 2015). Alternatively, tumor cells can bind to macrophages, neutrophils or platelets in the circulation and these cells mediate the adhesion of tumor cells to the endothelium acting as a bridge among tumor and endothelial cells (Evani et al., 2013; Reymond et al., 2013).

The evidence strongly suggests an stimulatory role of NO in tumor cell adhesion: NOS inhibitors blocked small cell lung carcinoma adhesion to the endothelium treated with pro-inflammatory cytokines (Vidal et al., 1992). NO enhanced fibrosarcoma cell adhesion and invasion through HUVEC monolayers, increasing ICAM-1 and ELAM-1 expression (Yudoh et al., 1997). *In vivo*, NO caused squamous carcinoma cell binding to the hepatic microcirculation (Scher, 2007). Breast circulating tumor cells MDA-MB-231 adhered to endothelial sites with high NO concentration (Zhang et al., 2016). *Rhus coriaria*, a medicinal plant that in part inhibits NO pathway decreases the adhesion and transmigration of MDA-MB-231 cells to endothelial cells activated with TNF-α (El Hasasna et al., 2016). The mechanisms that increase tumor cell adhesion to endothelium have not been studied in detail but are probably the same already described for leukocyte adhesion involving NF-κB transcriptional regulation, traffic and clustering of adhesion proteins. Additionally, another transcription factor, HIF-1α expressed in endothelial cells, is involved in extravasation of breast cancer cells through the expression of L1CAM adhesion protein that binds to tumor cells, and HIF-1α has been demonstrated to be activated by S-nitrosylation in others cellular contexts (Li et al., 2007; Zhang et al., 2012; Ehrenfeld et al., 2019).

As in leukocyte adhesion to endothelium, NO also shows a negative role in tumor cell adhesion: exogenously applied and endogenously generated NO inhibit melanoma cells adhesion to endothelium activated with lipopolysaccharide (Kong et al., 1996). The NO donor, CAP-NO, inhibited the basal and cytokine-stimulated adhesion of human colorectal cancer cells HT-29 to endothelial cells by inhibiting the expression of adhesion proteins ELAM-1, ICAM-1, and particularly VCAM-1 (Lu et al., 2014). NO donors inhibits the adhesion of MDA-MB231 cells to HUVEC stimulated by IL-1β and the transmigration of breast cancer cells across the lung microvascular endothelium (Kang et al., 2018; Stojak et al., 2018). This effect was mediated by a decrease in ICAM-1 expression. Similar to leukocyte adhesion, the differential effect of NO on tumor cell adhesion might depend on NO concentrations with low concentrations of NO increasing adhesion protein expression and adhesion of tumor cells, whereas higher NO concentrations, inhibiting protein adhesion expression and tumor cell adhesion (Sektioglu et al., 2016).

The effects of NO on adhesion protein expression not only affect tumor cell adhesion at the metastatic site but also infiltration of immune cells in the primary tumor, where it has been demonstrated that NO, produced by iNOS in M1 macrophages, mediates VCAM-1 expression in the endothelium leading to an improved infiltration of T-lymphocytes specific for the tumor, contributing to decrease tumor growth in a mouse melanoma model (Sektioglu et al., 2016).

After binding to tumor cells, adhesion proteins through their cytosolic tails interact with cytoskeletal proteins inducing changes in the shape of endothelial cells leading to destabilization of the endothelial barrier that facilitates tumor cell transmigration (Müller et al., 2001; Tichet et al., 2015). We have demonstrated that treatment of endothelial cells with conditioned medium from breast cancer cells and cytokines that are elevated in breast cancer patients induces S-nitrosylation of endothelial barrier proteins (p120, VE-cadherin and β-catenin) promoting phosphorylation and perturbation of the interactions among these proteins that leads to their internalization, which destabilizes the endothelial barrier (Marín et al., 2012; Guequén et al., 2016; Zamorano et al., 2019). Thus, better knowledge and understanding of NO biology in cancer is of paramount importance because it regulates not only expression of adhesion proteins that promote metastasis, but also controls the endothelial barrier to promote transmigration of tumor cells and metastasis.

## Conclusion

**FIGURE 5:**
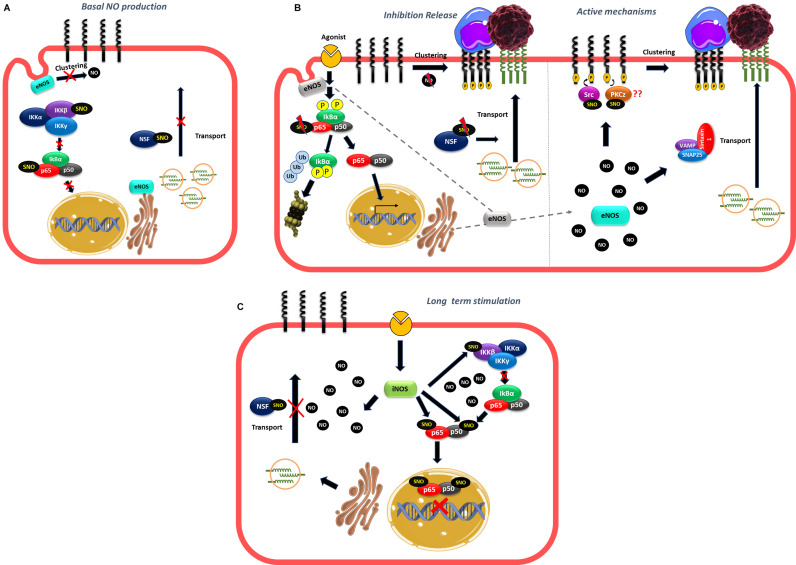
Role of NO in leukocyte and tumor cell adhesion. **(A)** Basal NO production produced by eNOS located to the Golgi and caveolae maintaining an anti-adhesive phenotype by inhibiting NF-κB activation, traffic of vesicles and clustering of adhesion proteins. **(B)** At short times of stimulation movement of eNOS away from Golgi and caveolae release the inhibition caused by NO to stimulate NF-κB activation and transcription of adhesion proteins, traffic of vesicles that contain adhesion proteins and clustering. At the same time, the rise in NO concentration in other subcellular locations may induce active mechanisms of adhesion such as formation of SNAREs complexes that stimulate vesicle traffic to the plasma membrane or to stimulate S-nitrosylation of targets such as Src and PKCz that induce clustering of adhesion proteins stimulating leukocyte and tumor cell adhesion. **(C)** At long times of stimulation, the high NO concentration induced by iNOS activation induce S-nitrosylation of NF-κB and NSF inhibiting the transcription of adhesion proteins and the vesicle transport of adhesion proteins to the plasma membrane.

The role of NO in leukocyte adhesion to endothelium has been controversial. Whether NO enhances or inhibits leukocyte adhesion depends on local NO concentration. It is reasonable to state that, in non-stimulated cells, NO concentration maintains an anti-adhesive phenotype at the endothelial cell membrane – inasmuch as inhibition of NOS causes increased leukocyte adhesion. This NO produced by eNOS prevents transport of vesicles to the cell surface, clustering of adhesion proteins and transcription of adhesion proteins by keeping NF-κB in an inactive state. However, an increase in eNOS-derived NO concentration to levels slightly above control is compatible with leukocyte adhesion through increasing cell surface transport, clustering of adhesion proteins and transcription of adhesion proteins via NF-κB activation. In addition, high NO concentration – most likely achieved through stimulation of iNOS – inhibits leukocyte adhesion due to S-nitrosylation of p50 and p65 in NF-κB and presumably IKKβ, which blocks entry of NF-κB to the nucleus leading to inhibition of adhesion protein synthesis. Similar to leukocyte-endothelium adhesion, NO also regulates tumor cell adhesion to endothelium probably through the stimulation of the same mechanisms described above as well as opening of the endothelial barrier, all of which enhance tumor cell extravasation and metastasis (Figure 5).

## Author Contributions

GA and TK collaborated in review literature and figures confection. PE collaborated in writing the manuscript and review literature. FS reviewed literature, wrote the manuscript, and reviewed figures. All authors contributed to the article and approved the submitted version.

## Conflict of Interest

The authors declare that the research was conducted in the absence of any commercial or financial relationships that could be construed as a potential conflict of interest.
